# Effects of Chemical and Natural Additives on Cucumber Juice’s Quality, Shelf Life, and Safety

**DOI:** 10.3390/foods9050639

**Published:** 2020-05-15

**Authors:** Mohamed T. El-Saadony, Mohamed F. Elsadek, Alaa S. Mohamed, Ayman E. Taha, Badreldin M. Ahmed, Ahmed M. Saad

**Affiliations:** 1 Department of Agricultural Microbiology, Faculty of Agriculture, Zagazig University, Zagazig 44511, Egypt; 2 Department of Community Health Sciences, College of Applied Medical Sciences, King Saud University, Riyadh 11362, Saudi Arabia; 3 Department of Nutrition and Food Science, Faculty of Home Economics, Helwan University, Helwan 11795, Egypt; Badreldin222@gmail.com; 4 Food Science Department, Faculty of Agriculture, Zagazig University, Zagazig 44511, Egypt; dr_alla88@yahoo.com; 5 Department of Animal Husbandry and Animal Wealth Development, Faculty of Veterinary Medicine, Alexandria University, Rasheed, Edfina 22758, Egypt; Ayman.Taha@alexu.edu.eg; 6 Biochemistry Department, Faculty of Agriculture, Zagazig University, Zagazig 44511, Egypt; ahmedm4187@gmail.com

**Keywords:** chemical additives, protein isolates, beverages storage, hyperbaric storage

## Abstract

Microbial contamination affects beverages’ lifetime, quality, and safety. Cucumber crops are seasonally spoiled because of the overproduction. The current study aimed to maximize the importance of natural preservatives and reduce the usage of artificial ones to prolong the cucumber juice’s storage life, enhance flavor, and control the microorganisms after protein isolate and organic acids supplementation. The additions included control (no addition), citric, benzoic acid, sodium salts, kidney bean pepsin hydrolysate (KPH), chicken egg protein isolate (CEPI), duck egg protein isolate (DEPI), and quail egg protein isolate (QEPI) as J-Control, J-Citric, J-Benzoic, J-sod. Citrate, J-sod. Benzoate, J-KPH, J-CEPI, J-DEPI, and J-QEPI, respectively. The antioxidant activity of these additives and juices was evaluated by DPPH radical scavenging activity. The antimicrobial activity, including antibacterial and antifungal activities, was evaluated by using disc assay and the radial growth of fungal mycelium, respectively. The phenolic compounds and flavonoids were estimated by a spectrophotometer as Gallic acid equivalent (GAE) and quercetin equivalent (QE), respectively. Moreover, chemical parameters such as pH, total soluble solids (TSS), Titratable acidity (TTA), and Vitamin C were evaluated by AOAC. Finally, the color properties were estimated by a spectrophotometer, using the Hunter method. KPH had higher significant (*p* ≤ 0.05) antioxidant activity (88%), along with antimicrobial activity. It significantly (*p* ≤ 0.05) reduced the growth of G+ and G− bacteria by 71–97% and 58–66% respectively. Furthermore, it significantly (*p* ≤ 0.05) inhibited the tested fungi growth by 70–88% and the other additives less than that. During the storage of cucumber juice for an interval of zero, two, four, and six months, the phenolic compounds and flavonoids were significantly (*p* ≤ 0.05) decreased. Consequently, the potential activity of the juice was reduced; in addition, pH and vitamin C were significantly (*p* ≤ 0.05) decreased during the storage period. Meanwhile, the TSS and Titratable acidity were significantly raised. As for color and sensory properties, J-sod. Benzoate, J-KPH, J-CEPI, and J-DEPI had significantly (*p* ≤ 0.05) high scores in color, taste, and flavor against the control. Generally, the usage of natural additives extends the cucumber juice’s lifetime and increased the manufacture of high-quality and valuable juice.

## 1. Introduction

The high moisture content in fruit and vegetable juices makes them highly susceptible to being spoiled by microorganisms [1] that can survive in acidic juices at normal temperature or refrigeration conditions, even when appropriately packaged. Moreover, physiochemical changes affect the safety and quality. All of these offensive changes may be prevented by supplementation of preservatives that maintain the nutrition value of juice, extend its lifetime, and keep it safe [2]. Recently, the consumer demand for foods with a long lifetime, high quality, and a suitable price has increased. Therefore, food producers and manufacturers are in quest of additives to increase food storage life, while maintaining nutrition value, quality, and safety. Many chemicals, such as nitrates, organic acids, and their salts, butylated hydroxytoluene (BHT) formaldehyde, and butylated hydroxyanisole (BHA) are effectively used as food preservatives, to reduce the microbial load and prolong food lifetime and vitality. Despite their antioxidant and antimicrobial activity [3,4], preservatives’ various undesirable effects on human health, including allergy, headache, asthma, hyperactivity, hypersensitivity, cancer, neurological damage, and dermatitis, have been investigated, [5,6,7]. Carocho et al. [8] confirmed that the extreme consumption of chemical additives is expounded to gastrointestinal, respiratory, dermatological, and neural opposing responses. Hence, consumers are increasingly concerned about the harmful effects of chemical preservatives and the preference for natural additives. Researchers have focused on producing natural preservatives that exhibit antioxidant and microbial activity for use in food processing [9,10]. Dauda et al. [11] evaluated the microbial load during watermelon juice storage over three months at cool conditions. They found that pure watermelon juice was highly vulnerable to microbial spoilage because of preservatives’ absence, and a large volume of microbial loads was recorded, but when they mixed the juice with serendipity berry extract as a preservative, the load was highly reduced and the storage life extended.

Cucumber (*Cucumis sativus* L.) is a member of Cucurbitaceae seasonal vegetable crops, which are native to India and cultivated all over the world [12]. During the harvest season, a large amount of cucumber spoils due to overproduction. This problem can be minimized by saving the cucumber as a drink or juice as functional beverages. In Central Asia, people drink cucumber juice on hot days, for recovery. Cucumber juice has health benefits for skin, nails, and hair; it maintained an ideal weight and cure some kidney disease and blood pressure issues. The cucumber is distinguished by its high content of water and satisfies all appetites [13]. In some regions of the developing countries, refrigeration storage is difficult to use due to the high costs of operating and the deficiency of electricity supplies. Alemu and Girma [14] developed a novel technique for food storage called “Hyperbaric storage”, which is usually not higher than 100 MPa at ambient or low temperature, for up to months. 

Recent studies showed that the storage with low pressure at room temperature can be an effective technique to fruit and vegetable juices’ storage, as pressure inhibits the microbial content of fresh juice, besides enhancing sensory characteristics and the quality of the juice. Reports indicated that low-pressure storage prolonged vegetable and fruit juices’ shelf life; it also reduced energy costs more than refrigeration storage [15,16,17,18]. Lemos et al. [19] preserved watermelon juice with low pressure (50–75 MPa), at room temperature, for two months. The usage of natural antimicrobial and antioxidants, such as herbal extracts and essential oils, improved capacity and safety in the food industry [20,21]. Moreover, peptides and antimicrobial proteins (AMPs) are responsible for the immunity of most organisms. Because of their activity and stability in foods and drinks, they represent natural preservatives [22]. Various protein hydrolysates have been applied as antioxidants and microbes in meat products [23,24]. Saad et al. [25] reported that kidney bean protein hydrolysate improved minced meat quality, shelf life, and safety. Wan and Xu [26] confirmed that whey protein isolates improved sensory and physical–chemical properties of a complimentary beverage. Organic acids exhibit antioxidant properties [3], especially dilute solutions, but they do not affect the sensory properties of food in, the sense that they do not affect the sensory properties of the carcasses [27]. 

This current study aimed to (i) investigate the effect of natural additives (different egg-white protein isolates; chicken, duck, and quail egg protein isolates; and kidney bean protein hydrolysate) and chemical additives (citric, benzoic acids, and their sodium salts) on cucumber-juice flavoring and preservation at room temperature, with low pressure, for an interval of zero, two, four, and six months; (ii) estimate the antioxidant and antimicrobial activities of the protein isolates and hydrolysates; (iii) identify the phenolic compounds and flavonoids in protein isolates and hydrolysates; and (iv) evaluate the sensory properties of juice during the preservation period. 

## 2. Materials and Methods

### 2.1. Materials

Cucumber (*Cucumis sativus*) and kidney bean *(Phaseolus vulgaris) L.* seeds were purchased from the local market. Chicken, duck, and quail eggs were obtained from a private farm (Zagazig City, Egypt). Proteins’ isolates were prepared at Biochemistry Department, Faculty of Agriculture, Zagazig University, Egypt. Pepsin enzyme, DPPH, Gallic acid, quercetin, benzoic acid, sodium benzoate, citric acid, sodium citrate, and nutrient agar media (lab-lemco powder) were purchased from Sigma (Ronkonkoma, NY, USA), MacConkey agar (Heywood, UK). The G+ bacteria (*Listeria monocytogenes* ATCC 15313, *Bacillus cereus* ATCC 11778, and G− bacteria (*E. coli* ATCC 25922, and *Pseudomonas aeruginosa* ATCC 27853) were purified active strains. Fungi strains, *Aspergillus niger*, *Penicillium notatum*, and *Fusarium solani* were obtained from the Cairo MIRCEN, Ain Shams University, Cairo, Egypt.

### 2.2. Methods 

#### 2.2.1. Preparation of Protein Isolates

##### Preparing of Pepsin Kidney Bean Protein Hydrolysate (KPH)

White kidney bean seeds were milled. The obtained flour was defatted with hexane (1:3 *w*/*v*) and then dried, in the oven. Total protein was isolated from five grams of defatted white kidney bean flour [28]. The lyophilized total protein isolate was dissolved in phosphate buffer pH two and hydrolyzed by pepsin enzyme (E:S ratio 1:200, *w*/*w*), at 37 °C, for three hours. The enzyme was deactivated in warm water at 90 °C for fifteen minutes. The supernatant was obtained by centrifugation of hydrolysate at 4000× *g* for thirty minutes, lyophilized, and then kept.

##### Preparation of Chicken, Duck, and Quail Egg White Protein Isolate (CEPI, DEPI, and QEPI)

The chicken, quail, and duck egg whites were diluted [29] with some modification, with water (1:3 *w*/*v*), and were stirred for thirty minutes and centrifuged for 20 min undercooling (15,000× *g*). The obtained supernatant was precipitated with 10% polyethylene glycol (PEG) 4000 and centrifuged under cooling at 15,000× *g* for twenty minutes. The residues were dissolved in 50 mM Tris-HCl, 200 mM NaCl, and 5 mM CaCl_2_, pH 7.8 (TBS-Ca). After leaving at 4 °C overnight, the mixture was centrifuged (4 °C, 15,000× *g*, 20 min). The precipitates were washed with TBS-Ca, homogenized with TBS buffer with ten mM EDTA, pH 7.8, and kept for thirty minutes at 4 °C. Then they were centrifuged at (4 °C, 1400× *g*, 20 min), and the supernatant was collected and adjusted to pH 5 with HCl. Following further centrifugation (4 °C, 1400× *g*, 20 min), it was dialyzed against 20 mM Tris-HCl, 50 mM NaCl (pH 8.0). The dialysate fractions were eluted on the Q Sepharose column by the gradual concentration of NaCl solution (0.1 to 0.6 molar). The chicken, duck, and quail egg protein’s isolates were diluted with NaCl (0.35 to 0.45 M) and then freeze-dried.

#### 2.2.2. Processing of Cucumber Juice Supplemented by Preservatives

The fresh cucumber was washed, cleaned, and processed in a Braun blender (Blender mixer Type 441), resulting in juice. The juice was heated at 83 °C for 2–3 min, with a few pressures 75 MPa “hyperbaric preservation” [17,19,30,31] in HIRAYAMA HG-SERIES autoclave (Concord, CA 94520, USA), and immediately cooled-down. Table 1 shows the juices constitutes by the Abbe Refractometer. The prepared juices were packed into sterilized bottles (350 mL) and divided into four groups, each of which included nine bottles, one for control and the others for juices supplemented with chemical and natural additives. The bottles were capped and tightly sealed and stored at room temperature for six months. The following analyses were carried out at intervals of preservation (0, 2, 4, and 6 months).

#### 2.2.3. Chemical Analysis

##### Estimation of Physiochemical Parameters

Titratable acidity of juices was calculated as citric acid (mg/mL) at the storage period of 0–6 months, at room temperature, according to standard method 942.15. Additionally, juice pH was assessed for the same samples by pH meter. Total soluble solids (TSS %) was determined by using an Abbe Refractometer (WZS portable refractometer, China). A few drops of the juice were mounted on the tip of the refractometer, and readings were taken [32]. Vitamin C was determined according to Ranganna [33]. A total carbohydrate was estimated according to the Chaplin [34] method. Then, 200 μL of hydrolysate sample and glucose standard (0, 20–100 μg/mL) was added to 200 μL of phenol (5%) and 1 mL of concentrated sulfuric acid. After thirty minutes, the OD was estimated at ƛ 490 nm. The concentration of total sugars in cucumber juices was calculated by using the linear equation in the glucose standard curve:

y = 0.0053x − 0.0193

R^2^ = 0.9884

where y is the absorbance, and x is glucose concentration (μg/ml).

##### Total Phenolic Compounds (TPC)

Total phenolic compounds (TPC) was assessed in cucumber juices supplemented with chemical preservatives and isolated proteins as GAE (0, 200–1000 μg/mL), following the Folin–Ciocalteu method [35], according to the equation of the Gallic acid standard curve:

y = 0.001x + 0.1369

R² = 0.9987

where y is the absorbance, and x is Gallic acid concentration (μg/mL).

##### Total Flavonoids

Three mL aliquot of 10 g/L AlCl_3_ ethanoic solution was added to 0.5 mL of each juice supplemented with chemical preservatives and isolated proteins, the mixtures were then incubated for an hour, at room conditions [36]. The absorbance was estimated at 430 nm. Total flavonoids in samples was measured as QE (0, 20–100μg/mL), using the quercetin acid standard curve equation.

y = 0.0023x − 0.0035

R^2^ = 0.9989

where y is the absorbance, and x is the quercetin concentration (μg/mL).

##### DPPH Radical-Scavenging Activity

DPPH radical-scavenging activity was followed in milk samples, as an indicator of antioxidant activity [37]. An aliquot (100 μL of each sample) was added to 1 mL of 1 mL DPPH in ethanol and incubated at room temperature for thirty minutes [38], before measuring the color absorbance at 517 nm against a control. The percentage of antioxidant activity of free radical DPPH was calculated as follows:

$$Radical\;scavenging\;activity\;\left(\%\right)=\frac{\left(A\;control-A\;sample\right)}{Acontrol}\times 100$$

where *A_control_* is the control absorbance, and *A_sample_* is sample absorbance, i.e., DPPH reaction absorbance.

#### 2.2.4. Color Measurements

The color of cucumber juices was measured by using a spectrophotometer (Hunter Lab, Color Flex EZ’s 45°/0°, Reston, VA, USA). CIELAB system: L* (lightness–darkness), a* (redness–greenness), b* (yellowness–blueness), H (Hue angle), C* (Chroma), WI (whiteness index), and differences values were measured. The instrument was calibrated by using a standard white title: X = 72.26, Y = 81.94, Z = 88.141, L* = 92.46, a* = −0.86, and b* = −0.16 [39].

$$\mathrm{Hue}\;\mathrm{angle}\;\left(H\right)={\;\tan}^{-1}\left(\frac{b*}{a*}\right)$$

$$\mathrm{Chroma}\;\left(C*\right)={\left({a*}^{2}+{b*}^{2}\right)}^{\frac{1}{2}}$$

WI_H_ = L − 3b (Hunter L, a, b C/2) = 10 (Y − 21)^1/2^ (Y − 0.847 Z) Y^1/2^ (C/2)

#### 2.2.5. Sensory Evaluation

Eighty members (faculty staff and students) from the Food Science Department, Faculty of Agriculture, Zagazig University, Egypt, evaluated the sensory properties of cucumber juices, (control+ eight juice with different additives), using a scorecard for each sensory attributes (color, odor, flavor, taste, and overall acceptability), using a 9-point Hedonic scale, whereby the scores ranged from dislike extremely (1) to like extremely (9) [40]. The room was illuminated with white light, and each session continued for two hours. Water was provided to each panelist for mouth-rinsing after testing each product, to avoid the carry-over effect.

#### 2.2.6. Microbial Analysis

##### Antibacterial Activity

Antibacterial activity was estimated [41,42]. Paper discs saturated with 30 μL of each additive at different concentrations (0, 50, 100, and 200–1000 mg/mL) were then added to Petri dishes containing nutrient agar infected with pathogenic microorganisms, *the G+ bacteria (L. monocytogenes* and *B. cereus)*, and *G− bacteria (E. coli* and *Ps. aeruginosa)* incubated at 37 °C for 24 h. The developed inhibition zones (mm) were manually measured by using a transparent ruler. The negative control was disc-saturated with distilled water. Minimum inhibitory concentration (MIC) was estimated as the lowest concentration and showed a clear zone on MHA plates.

Turbidity (A600) assay was used to determine the extent of the bacterial growth in nutrient broth media suspensions during 24 h of incubation. The MIC of each sample was added to a tube containing 100 μL of pathogenic bacteria in 10 mL nutrient broth, incubated at 37 °C and measured every six hours, before recording the turbidity compared with control.

##### Microbial Count

Total viable count and coliform bacterial count in cucumber juices supplemented with chemical preservatives and isolated proteins at 0.2% (*w*/*v*) were performed during preservation periods (0–6 months), at room temperature, by using the pour plate technique [43]. First, 1 mL (*v*/*v*) of the sample was diluted with one-fold of 2% sterile sodium citrate solution, to prepare a suspension. Then, 1 mL of the suspension was used for the serial dilution of between 10^−1^ and 10^−5^. After that, 1 mL of each dilution was placed in sterile disposable Petri dishes (sterilin) in triplicates. At about 44 to 50 °C, the number of different bacteria was determined by using specified media [44,45,46]. Colon bacteria were counted on MacConkey agar and brooded at 37 °C for 24 h. The total viable count (TVC) on Agar media was counted and incubated at 25 m, for a period of 72 h. Microbiological results were converted to logarithms (CFU/g).

##### Antifungal Activity

The inhibition action of the chemical and natural additives against three fungal species were obtained from the Department of Agricultural Microbiology, Faculty of Agriculture, Zagazig University, Egypt [47,48]. First, 5 mL of each additive, at different concentrations (0, 50, 100, 200, 400, 800, and 1000 μg/mL), was poured into potato dextrose agar (PDA) medium in Petri dishes and then protected at 28 °C for seven days. After 24 h of incubation, mycelia disk (5 mm) was carefully picked from the edge of fungal cultures and placed in the center of each Petri dish containing the additives. The PDA plates without any addition or water were prepared as negative and positive controls, respectively. The fungal mycelium’s radial growth was recorded (cm). The minimum fungal concentration was estimated according to [49], by inoculating the contents of all the prepared fungi combined with the additives’ concentrations in test tubes, as prepared in case of an MIC test on new PDA tubes. All test tubes were nurtured at 28 °C, for 48 to 72 h.

#### 2.2.7. Statistical Analysis

The obtained data means were statistically analyzed by using Microsoft Office Excel (version 2019) and ANOVA variance single factor, at a probability level of *p* ≤ 0.05; multiple comparisons were carried out, applying the least significant difference (LSD).

## 3. Results and Discussion

Cucumber juice is considered a valuable and therapeutic beverage. It possesses different medicinal properties, such as antimicrobial, antioxidant, and anticancer properties [50].

### 3.1. Physiochemical Changes Analysis

#### 3.1.1. DPPH Radical-Scavenging Activity of Additives

**Figure 1:**
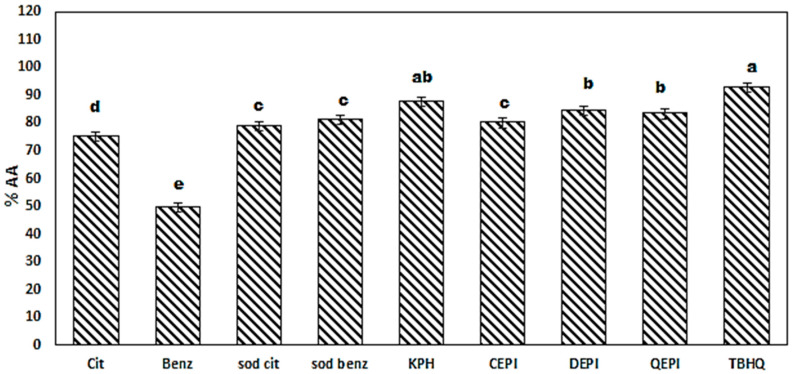
DPPH radical-scavenging activity of cucumber peel after 30 min compared with TBHQ. Mean with different letters above each column are significantly different (*p* ≤ 0.05).

Figure 1 shows the antioxidant activity of chemical additives from citric acid, benzoic acid, sodium citrate, and sodium benzoate, as well as natural ones from KPH to QEPI, pepsin kidney bean peptide, chicken egg protein isolate, duck egg protein isolate, and quail egg protein isolate. All preservatives were added at a concentration of 0.2% (*w*/*v*). KBH significantly (*p* ≤ 0.05) exhibits the highest radical-scavenging activity, with 88% compared to TBHQ 93%, with a relative increase ranging from 5% to 9% from DEPI, QEPI, and sodium benzoate.

The antioxidant activity of organic acid and their salts depends on the carboxyl group giving an electron to free radical and converting to less-reactive (stable) acyl free radical that can be reduced into organic acid again or oxidized to dehydro-organic acid [3]. Sarmadi and Ismail [51] and Afandi et al. [52] investigated the free-radical-scavenging action of protein isolates and hydrolysate with two mechanisms, hydrogen atom transfer (HAT) and single electron transfer (SET). They may act in parallel or with one dominating, according to protein isolate structure, wherein the aromatic amino acids convert radicals to stable molecules by donating electrons, but keeping their own stability. Hydrophobic amino acids improve peptides’ solubility in lipids through hydrophobic side chains. Meanwhile, essential and acidic amino acids act as metal ion chelators and proton donners through their COOH and NH_2_ side chain.

#### 3.1.2. Antioxidant Activity of Cucumber Juice

**Figure 2:**
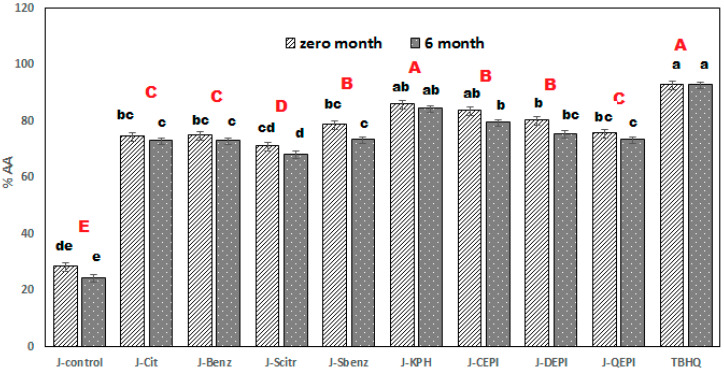
Antioxidant activity changes of cucumber juices during the storage period. Means with different lowercase letters that sit above columns are significantly different during storage, with different red capital letters, and significantly different between samples (*p* ≤ 0.05). J-control, control cucumber juice; J-citric, cucumber juice supported with citric acid, J-benzoic, cucumber juice supported with benzoic acid; J.s-citrate, cucumber juice supported with sodium citrate; J.s-benzoate, cucumber juice supported with sod. Benzoate; J-KPH, cucumber juice supported KBH; J-CEPI, cucumber juice supported with CEPI; J-DEPI, cucumber juice supported with DEPI; J-QEPI, cucumber juice supported with QEPI.

Figure 2 shows the antioxidant activity changes of cucumber juices during the storage period of zero, two, four, and six months period. The antioxidant activity of cucumber juices significantly (*p* ≤ 0.05) decreased. There was no significant decrement in J-KPH, with the smallest relative decrease about 1.5%; the antioxidant activity relatively reduced from 7% to 14% for juices from J-QEPI to J-control. Klimczak et al. [53] reported that the decrease in antioxidant activity might be linked to a reduction in total phenolic content and vitamin C during storage. Protein–phenolic complexes’ formation may affect the physical and chemical properties of the protein. Moreover, the phenolics–proteins binding due to blocking some amino acid side chains possibly increased the activity. Furthermore, the protein–phenolic complex may also increase the bioaccessibility and activity of phenolics [54,55,56,57,58].

#### 3.1.3. Phenolic Compounds and Flavonoids

Table 2 shows that the significant (*p* ≤ 0.05) relative decrease of total phenolic content ranged from 9% to 23% GAE μg/mL after six months. Besides, flavonoids were significantly (*p* ≤ 0.05) decreased from 38.515–29.727 to 29.764−22.212 QE μg/mL after six months, for juices. Vallverdu-Queralt et al. [59] found a decrease in total polyphenol content of tomato juices after three, six, and nine months of storage. Consequently, Kaur et al. [60] showed a significant reduction of phenolic compounds during the six-month storage of cucumber juice supplemented with chemical additives. The protein isolates significantly (*p* ≤ 0.05) maintained the fluids more than the chemically treated sample. The lowest decrement was found in J-KPH, with about 9% relative decrease.

#### 3.1.4. Chemical Parameters

**Figure 3:**
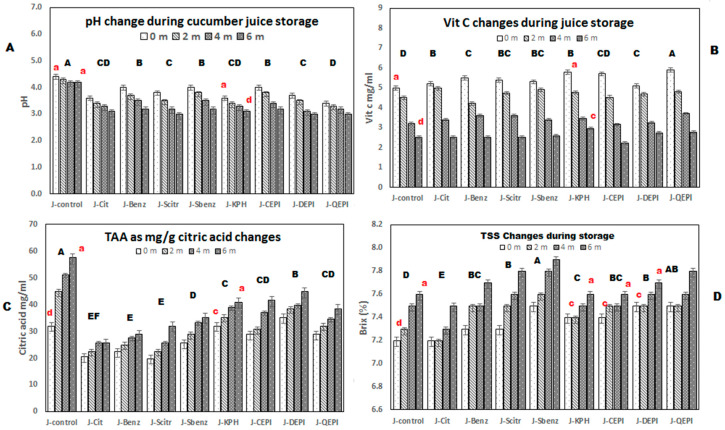
Chemical parameters’ changes during cucumber juices storage for zero to six months, at room temperature. Mean with different red lowercase letters that above columns are significantly different during storage, with different capital letters, significantly different between samples (*p* ≤ 0.05). (**A**) pH change during cucumber juice storage; (**B**) Vit C change during juice storage; (**C**) TAA as mg/g citric acid changes; (**D**) TSS changes during storage.

Table 3 shows that total sugars significantly (*p* ≤ 0.05) decreased from the range of 278.81–170.70 mg for J-control–J-QEPI, on the day of preparation, to the range of 95.79–52.40 mg after six months, a relative decrease of 65–70%. Juice acidity was increased because of total sugar in juice analyzed into simple sugar by the fermentative effect of acid-producing bacteria in agreement with Sivakumar [61]. Although TSS not significantly increase because of increments of simple sugars (Figure 3D), Kausar et al. [62] observed that TSS increased (15.49–16.09%) during the storage of cucumber-melon drink [50] and cucumber–mint drink. Similar results were noticed in watermelon juice blended with ginger obtained by [63]. Kinh et al. [64] reported an increase in soluble content of apple pulp during storage when preserved with chemical preservatives. Figure 3A shows significant (*p* ≤ 0.05) decreasing of pH value, from 4.4 to 3.6, in all samples after six months, with a relative decrease of about 25–30%. Besides that, the results indicated that pH changes occur less in natural additives than in chemical and control. The pH decreased because of increasing in acidity. Aderinola et al. [65] observed a decrease in pH values and an increase in TTA during the storage of carrot–cucumber juice; these changes might occur due to the fermentation of sugar present in the juice. The acidity in juices J-control to J-Sod-Benz significantly (*p* ≤ 0.05) increased more than fluids supplemented with protein isolates (Figure 3C), and the changes were significant at least in J-KPH, J-CEPI, and J-DEPI. As per results, natural additives > chemical additives have a significant (*p* ≤ 0.05) effect on Vitamin C content of cucumber juice. On the storage debut, the Vitamin C content in cucumber juices ranged from 5 to 5.3 mg/100 g, respectively. The values significantly (*p* ≤ 0.05) faded, as heat treatment destroys, at the end of the storage period to 2.5–2.3 (Figure 3B). Francis et al. [66] detected the decrement in vitamin C of watermelon juices blended with sodium benzoate and lime; this degradation might be due to the high sensitivity of light, oxygen, heat, and enzymatic or non-enzymatic oxidation.

### 3.2. Color Measurements and Sensory Evaluation

Table 4 showed the juices color parameters, where the L* non-significant decrease from 29.24 for control to 28.7 at the end of storage, with low relative decrease 3%, nearly no change in a, and slightly increase in b from 11.27 to 11.29. The significant (*p* ≤ 0.05) best color, according to L* results, was juice five, supplemented with sodium benzoate. Besides, J-KPH increased in L* from 28.05 to 29.03 with a relative increase of 4%, decreased in a* from −3.2 to −3.4, and showed no change in b*. J-sod-citrate was significantly less white, and other color parameters, L* 22.8, a* 0.44, and b* 11, increased after six months to 23, 0.76, and 12. Tomato juice with Na benzoate seems to be more stable than the other preservatives during six months of storage and was less off-color and developed less turbidity [60,67].

Packing material, storage time, temperature, pressure, and microbial contamination intensely alter juice flavor. Due to all of these factors, furfural increased during storage, as an indicator of acid hydrolysis of alcohols and terpenes. The sensory observations during the storage period are shown in Table 5, and it is seen that there is a slight significant (*p* ≤ 0.05) variation in color among the nine samples during the storage period. The color was found to be yellow/dark yellow/reddish on the day of preparation. The color faded and flavor declined gradually with the increase of storage period at room conditions. Juices five, six, seven, and eight were the best in color stability and overall acceptability, and juice nine was the worst-rated [65].

### 3.3. Microbial Analysis

#### 3.3.1. Antibacterial Activity of Additives

Table 6 shows the inhibition zones diameter (IZD) of chemical and natural preservatives at MIC levels. The MIC of G+ bacteria was 50 μg/mL for additives; citric, sod-citrate, sod-benzoate, KPH, and DEPI, and 100 for the rest; however, the MIC for G− bacteria was 50 for KPH and DEPI; 100 for sod-benzoate, CEPI, and QEPI; 200 for citric and sod-citrate; and 400 for Benzoic, as shown in Table 6. The Gram-positive strain showed higher vulnerability values than the Gram-negative one. *B. cereus* and *L. monocytogenes* recorded IZD ranging from 20 to 33 mm and 21 to 30 mm for sodium benzoate and KBP, respectively. *E. coli* and *Ps. aeruginosa* have the lowest IZD for tested additives (11–18 mm) for citric acid, sodium citrate, and benzoic acid. KBP had a significantly higher (*p* ≤ 0.05) IZD, and QPI had the lowest. The lower zones of inhibition observed in the G− organisms compared to the G+ organisms due to the peptidoglycan-containing periplasmic space and the outer membrane lipopolysaccharide layer of G− bacteria acts as a barrier, preventing the penetration of numerous environmental substances [68], including antimicrobial substances, into the organism. The periplasmic space is also known to contain enzymes capable of breaking down foreign molecules attempting to gain entry into the microorganism [69,70].

#### 3.3.2. Antifungal Activity

Table 6 showed the inhibition of fungal radial growth (Cm) of chemical and natural preservatives, where the lowest MFC ranged from 800 to 1000 μg/mL for all fungal strains. KPH had the significant highest (*p* ≤ 0.05) values in fungal growth inhibition, followed by sodium benzoate, DEPI, and CEPI, as shown in Table 6. The antifungal action of organic acids and protein isolates is reported according to [4,69,70,71].

#### 3.3.3. Bacterial Growth Curve

**Figure 4:**
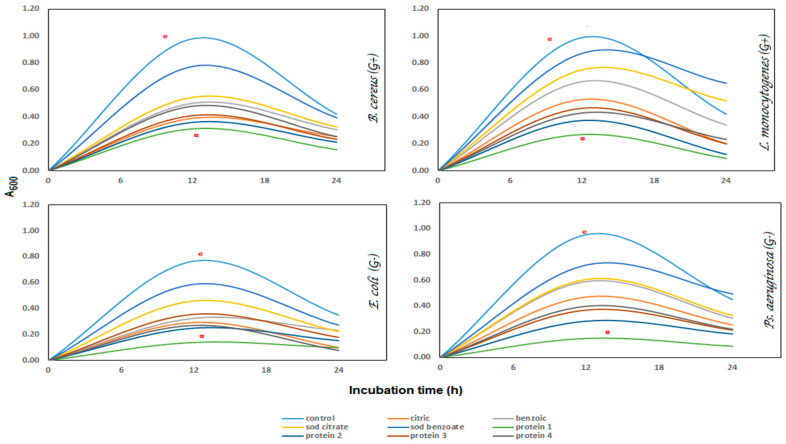
Growth curve of G^+^ and G^−^ bacteria in the presence of additives MIC levels.

The growth curves of the tested bacteria reached the highest turbidity after about 16 h at 37 °C in *E. coli* and *Ps. aeruginosa*, but after 12 h in *B. cereus* and *L. monocytogenes.* KBH had significant (*p* ≤ 0.05) values in reducing the growth of Gram-positive bacteria by 71% and 79%, as well as Gram-negative bacteria by 58% and 66%, for *B. cereus, L. monocytogenes, E. coli*, and *Ps. aeruginosa*, respectively. Generally, chemical additives reduced the growth of *B. cereus* by 10–45%, *L. monocytogenes* by 18–58%, *E. coli* by 10–38%, and *Ps. aeruginosa* by 20–44%.However, the natural ones reduced the G+ bacteria growth by 55–71% and 53–79%, as well as by 41–58% and 49–66% for G− bacteria, as shown in (Figure 4). The antimicrobial mechanism of organic acid penetrated the microorganisms’ cell membrane, decreased cell pH, and controlled the processes of metabolism, especially the synthesis of ATP, RNA, protein, and DNA replication [4]. Most of the positively charged peptides are electrostatically bound to negatively charged compounds on the bacterial cell wall, leading to cell wall destruction [71,72,73]. Furthermore, the peptide hydrophobicity plays an essential role in disturbing the bacterial cell membrane and cell wall. MIC was determined from chemical and natural additives against all experimental bacterial strains.

#### 3.3.4. Bacterial Load in Cucumber Juice during Storage

Table 7 showed that the highest total bacterial count obtained in control during storage ranged from 2.7 to 5 (log CFU/mL) at room temperature, from two to six months. Meanwhile, the bacterial load significantly (*p* ≤ 0.05) decreased in J-citric and J-benzoic decreased from 2.7 to 2.5, and 2.6 respectively, with about 50%. On the other hand, the bacterial load of J-KPH, J-CEPI, and J-DEPI significantly (*p* ≤ 0.05) decreased with a relative increase of about 60%, because of the antimicrobial potential of protein isolates [71,72,73]. Similar results were obtained by Habib and Iqbal [30], who observed the least TVC in mixed cucumber–tomato–pumpkin juice, especially in the blend percentage 5%–7%–9%.

## 4. Conclusions

The study was able to establish the KBH, and Sod-Benzoate has a significant effect on the lifetime and sensory properties of the treated juice samples. It could be concluded that cucumber juice blended/enhanced with KPH and sodium benzoate (0.2%) was rated higher when compared to other samples. The addition of KBH to the cucumber juice maintained the storage life of the juice for six months, at room temperature, with 50–75 MPa, thus making it an available meal, ready to serve, and a refreshing drink with a good nutritional, medicinal, and caloric value. The results of this research work confirmed both the vulnerability of pure cucumber juice to a microbial attack due to its high moisture content and the preservative potentials of natural additives, especially KBH, because of their antioxidant and antimicrobial activity. It was observed that KBH had two significant roles in the juice samples; that of a flavor (natural) and a preservative. We recommended the utilization of natural additives because of their safety, unlike chemical ones. Finally, this preservation method protected the cucumber crop from spoilage and increased the manufacture of high-quality and valuable juice.

## Figures and Tables

**Table 1:** The amount of cucumber juice ingredients preserved via chemical and natural preservatives.

Constitutes	Juices in Group								
	J-Control	J-Citric	J-Benzoic	J-Sod-Citrate	J-Sod-Benzoate	J-KPH	J-CEPI	J-DEPI	J-QEPI
Juice (mL)	200	200	200	200	200	200	200	200	200
Citric acid (g)	0.3	0.3	0.3	0.3	0.3	0.3	0.3	0.3	0.3
Sugar (g)	10.4	10.4	10.4	10.4	10.4	10.4	10.4	10.4	10.4
Citric (g)	-	0.3	-	-	-	-	-	-	-
Benzoic (g)	-	-	0.3	-	-	-	-	-	-
Sod citrate (g)	-	-	-	0.3	-	-	-	-	-
Sod-benzoate (g)	-	-	-	-	0.3	-	-	-	-
KBH (g)	-	-	-	-	-	0.3	-	-	-
CEPI (g)	-	-	-	-	-	-	0.3	-	-
DEPI (g)	-	-	-	-	-	-	-	0.3	
QEPI (g)	-	-	-	-	-	-	-	-	0.3

KPH, kidney bean protein hydrolysate; CEPI, chicken egg protein isolate; DEPI, duck egg protein isolate; QEPI, quail egg protein isolate; J-control, control cucumber juice; J-citric, cucumber juice supported with citric acid, J-benzoic, cucumber juice supported with benzoic acid; J.s-citrate, cucumber juice supported with sodium citrate; J.s-benzoate, cucumber juice supported with sod. Benzoate; J-KPH, cucumber juice supported KBH; J-CEPI, cucumber juice supported with CEPI; J-DEPI, cucumber juice supported with DEPI; J-QEPI, cucumber juice supported with QEPI.

**Table 2:** Changes in total phenolic compounds and flavonoids during period storage.

Sample	TPC		TFC	
	Storage Period (Month)			
	0	6	0	6
J-control	532.74 ± 0.7 f	409.10 ± 1.1 e	38.515 ± 1.2 e	29.764 ± 0.9 e
J-Citric	1228.03 ± 0.5 b	1111.10 ± 0.9 b	47.000 ± 0.9 d	44.872 ± 1.2 c
J-Benzoic	933.46 ± 0.6 d	739.10 ± 0.8 c	45.788 ± 0.8 d	43.714 ± 1.3 c
J-Sod-Benzoate	1284.39 ± 0.25 b	1028.10 ± 1.3 b	34.879 ± 0.6 e	33.290 ± 0.8 d
J-Sod-Citrate	1133.81 ± 0.1 c	904.10 ± 1.2 b	45.182 ± 0.7 d	43.135 ± 0.8 c
J-KPH	1417.96 ± 0.9 a	1268.10 ± 0.9 a	120.030 ± 1.3 a	99.212 ± 0.5 a
J-CEPI	1166.60 ± 0.4 c	931.10 ± 0.7 b	100.636 ± 1.6 b	96.125 ± 0.25 a
J-DEPI	1088.89 ± 0.22 d	867.10 ± 1.5 c	54.879 ± 1.9 c	52.401 ± 1.2 b
J-QEPI	770.74 ± 1 e	605.10 ± 1.6 d	29.727 ± 2 f	22.212 ± 2.3 e

Mean ± SD. Cucumber-juice means in the same column with different letters are significantly different (*p* ≤ 0.05). J-control, control cucumber juice; J-citric, cucumber juice supported with citric acid, J-benzoic, cucumber juice supported with benzoic acid; J.s-citrate, cucumber juice supported with sodium citrate; J.s-benzoate, cucumber juice supported with sod. Benzoate; J-KPH, cucumber juice supported KBH; J-CEPI, cucumber juice supported with CEPI; J-DEPI, cucumber juice supported with DEPI; J-QEPI, cucumber juice supported with QEPI.

**Table 3:** Changes of total sugars during storage for zero and six months, at room temperature.

Sample	Storage Period (Month)			
	0	2	4	6
J-control	278.81 ± 0.5 b	104.09 ± 0.3 c	89.19 ± 0.2 b	95.79 ± 0.1 b
J-Citric	259.38 ± 0.2 b	91.64 ± 0.8 d	67.49 ± 0.4 c	85.23 ± 0.3 c
J-Benzoic	207.68 ± 0.8 c	76.55 ± 0.9 e	54.28 ± 0.6 d	67.87 ± 0.2 d
J-Sod-Benzoate	106.36 ± 0.9 f	49.19 ± 0.7 f	30.13 ± 0.2 e	35.79 ± 0.7 f
J-Sod-Citrate	298.81 ± 0.7 a	192.77 ± 1.2 a	143.53 ± 0.8 a	133.53 ± 0.9 a
J-KPH	121.45 ± 0.1 e	95.79 ± 1.3 d	66.50 ± 0.6 c	50.20 ± 1 d
J-CEPI	115.04 ± 0.13 f	91.08 ± 0.5 d	38.43 ± 0.7 e	33.53 ± 1.2 f
J-DEPI	129.38 ± 0.2 e	107.87 ± 0.4 c	55.09 ± 0.5 d	45.25 ± 1.3 e
J-QEPI	170.70 ± 0.3 d	155.04 ± 0.1 b	58.62 ± 0.5 d	52.40 ± 0.9 d

Mean ± SD. Cucumber-juice means in the same column with different letters are significantly different (*p* ≤ 0.05).

**Table 4:** Changes in color parameters during juices’ storage.

**Juices**		**0 Months**						
	**L**	**a**	**b**	C *	**h**	**WI**	**Differences**	**b/a**
J-control	29.24 b	−3.3 c	11.27 b	11.74	−73.7	28.27	0	−3.42
J-Citric	27.77 d	−2.91 b	10.81 c	11.19	−74.91	26.91	1.59	−3.71
J-Benzoic	28.61 c	−2.81 b	11.63 b	11.96	−76.42	27.61	0.88	−4.14
J-Sod-Benzoate	22.8 e	0.44 a	11 c	11.01	87.71	22.02	7.45	25
J-Sod-Citrate	32.44 a	−3.69 c	11.64 b	12.21	−72.39	31.35	3.24	−3.15
J-KPH	28.05 c	−3.23 c	10.46 c	10.95	−72.85	27.22	1.44	−3.24
J-CEPI	28.45 c	−3.33 c	13.32 a	13.73	−75.96	27.14	2.2	−4
J-DEPI	27.63 d	−2.67 b	12.96 a	13.23	−78.35	26.43	2.42	−4.85
J-QEPI	29.08 b	−3.92 c	13.56 a	14.12	−73.88	27.69	8.06	−3.46
**Juices**		**6 Months**						
	**L**	**a**	**b**	C *	**h**	**WI**	**differences**	**b/a**
J-control	28.7 b	−3.33 c	11.29 c	11.77	−73.56	27.73	0	−3.39
J-Citric	27.9 c	−2.81 b	10.9 c	11.26	−75.55	27.03	1.03	−3.88
J-Benzoic	28.9 b	−2.79 b	11.43	11.77	−76.29	27.93	0.59	−4.1
J-Sod-Benzoate	23 d	0.76 a	12 b	11.03	86.05	22.21	7.02	14.47
J-Sod-Citrate	33 a	−3.71	11.64 c	12.22	−72.33	31.9	4.33	−3.14
J-KPH	29.03 b	−3.4 c	10.46 d	11	−72.01	28.18	0.9	−3.08
J-CEPI	28.6 b	−3.4 c	13.65 a	14.07	−76	27.23	2.36	−4.01
J-DEPI	27.9 c	−2.5 b	12.99 b	13.23	−79.11	26.7	2.05	−5.2
J-QEPI	30 a	−8 d	13.71 a	15.87	−59.68	28.22	11.54	−1.71

Means in the same column with different letters are significantly different (*p* ≤ 0.05). L * (lightness–darkness), a * (redness–greenness), b * (yellowness–blueness), H (Hue angle), C * (Chroma), WI (whiteness index.

**Table 5:** Sensory properties of cucumber juices during the storage period.

Juices	0 Months			
	Color	Taste	Flavor	Overall Acceptability
J-control	8 c	8.5 a	8.3 b	8.27 b
J-Citric	8.5 b	8.7 a	8.5 a	8.57 a
J-Benzoic	8.4 b	8.2 b	8.9 a	8.50 a
J-Sod-Benzoate	7.5 c	8.9 a	8.8 a	8.70 a
J-Sod-Citrate	8.3 b	8.6 a	8.2 b	8.37 b
J-KPH	9 a	8.8 a	8.6 a	8.80 a
J-CEPI	8.1 c	7 c	7.5 c	7.53 c
J-DEPI	7.9 c	6.7 c	7.3 c	7.30 c
J-QEPI	7.9 c	7.1 c	7.1 c	7.37 c
	**6 Months**			
J-control	6.75 c	7.15 b	7 a	6.97 b
J-Citric	7.25 bc	7.35 b	7.2 a	7.27 a
J-Benzoic	7.15 bc	6.85	7.6 a	7.20 a
J-Sod-Benzoate	6.8	7.55 a	7.5 a	7.60 a
J-Sod-Citrate	8 a	7.25 b	6.9 b	7.07 b
J-KPH	7.75 b	7.45 a	7.3 a	7.50 a
J-CEPI	6.85 c	5.65 d	6.2 b	6.23 b
J-DEPI	6.65 c	5.35 d	6 b	6.00 b
J-QEPI	6.65 c	5.75 d	5.8 c	6.07 b

Means in the same column with different letters are significantly different (*p* ≤ 0.05).

**Table 6:** Antimicrobial activity of chemical and natural additives.

Addi-tives	Concen-tration	G+ bacteria		G− bacteria		Fungi		
		*B. cereus*	*L. monocytogenes*	*Ps. aeruginosa*	*E. coli*	*A. niger*	*P. notatum*	*F. solani*
**1**	0	-	-	-	-	-	-	-
	50	8E	9E	-	-	6A	7A	6A
	100	12	13	-	-	5	4	3
	200	16	17A	15	12	5	6	4
	400	17A	17	17A	13	4	3	5
	800	16	15	16	16A	3	1	1
	1000	16	12	14	11	1E	0F	0E
2	0	-	-	-	-	-	-	-
	50	-	-	-	-	4	2	3
	100	11D	14	-	-	4	5A	3
	200	15	15	-	-	6A	2	4A
	400	16	12	12C	11C	4	2	1
	800	14	14C	14	15A	3	2	1
	1000	17A	20A	16A	12	0E	1D	0E
3	0	-	-	-	-	-	-	-
	50	10E	11D	-	-	4A	5	4
	100	13	14	-	-	3	4	5A
	200	17A	18	16	13	4	6A	4
	400	16	17A	15	14	3	2	5
	800	15	16	18A	16A	2		1
	1000	17	14	15C	12C	1D	1E	0D
4	0	-	-	-	-	-	-	-
	50	12	14C	-	-	6A	7A	6A
	100	15C	17	8E	7E	4	5	4
	200	19	18	17	16	4	4	6
	400	18	19	20A	18A	2	5	5
	800	17	20	19	16	1	1	2
	1000	20A	21A	16	13	0E	1E	0E
5	0	-	-	-	-	-	-	-
	50	19C	17D	16C	14D	8A	7A	8A
	100	19	17	16	15	5	5	6
	200	20	18	16	15	4	4	6
	400	33A	30A	29A	27A	2	5	5
	800	30	26	25	23	1	3	2
	1000	29	25	23	21	0E	0E	0E
6	0	-	-	-	-	-	-	-
	50	-	-	-	-	6A	4A	5A
	100	14D	12D	16C	15D	5	4	5
	200	19	15	16	15	5	2	3
	400	29A	24A	29A	27A	4	1	3
	800	27	22	25	23	3	1	2
	1000	25	23	23	21	0E	0E	1D
7	0	-	-	-	-	-	-	-
	50	17C	15	14	13	5A	4	5A
	100	17	14D	13C	12D	3	5A	4
	200	29A	18	14c	11	5	3	4
	400	29	25A	25A	20	2	5	5
	800	28	24	23	21A	0D	0D	1
	1000	27	23	21	19	0	0	0F
8	0	-	-	-	-	-	-	-
	50	-	-	-	-	4	6A	4
	100	19C	17C	11D	10D	5	3	2
	200	20	18	14	11	6A	5	3
	400	33A	30A	27A	23A	4	6	5A
	800	30	26	22	21	2	4	3
	1000	29	25	23	21	1E	0E	1E

Cucumber juice bold means in the same column with different capital letters are significantly different (*p* ≤ 0.05). -: not detected; 1: citric acid; 2: benzoic acid; 3: sod-citrate; 4: sod-benzoate; 5: KPH; 6: CEPI; 7: DEPI; 8: QEPI.

**Table 7:** TVC and CBC in cucumber juices during the storage period at room temperature.

Juices	Log CFU/mL							
	0 Months		2 Months		4 Months		6 Months	
	TVC	CBC	TVC	CBC	TVC	CBC	TVC	CBC
J-control	1.2 ± 0.1a	ND	2.7 ± 0.2a	ND	3.5a ± 0.5	1.3a ± 0.3	5a ± 0.7	1.5a ± 0.2
J-Citric	0.9 ± 0.08b	ND	2.5 ± 0.19b	ND	2.9b ± 0.4	0.9b ± 0.4	4c ± 0.5	1.1b ± 0.3
J-Benzoic	1.2 ± 0.1a	ND	2.6 ± 0.18a	ND	2.95b ± 0.4	1.1a ± 0.3	4.5b ± 0.8	1.4a ± 0.5
J-Sod-Benzoate	1.1 ± 0.15b	ND	2.9 ± 0.3a	ND	2.89b ± 0.4	1.2a ± 0.3	4.7a ± 0.9	1.3a ± 0.4
J-Sod-Citrate	0.9 ± 0.09b	ND	2.5 ± 0.19b	ND	3.1c ± 0.2	0.8b ± 0.2	4c ± 0.5	0.9c ± 0.1
J-KPH	0.5 ± 0.03d	ND	2.3 ± 0.1c	ND	2.8c ± 0.3	0.7c ± 0.25	4c ± 0.5	0.9c ± 0.1
J-CEPI	0.8 ± 0.04c	ND	2.4 ± 0.1b	ND	2.89 ± 0.4	0.8b ± 0.2	4c ± 0.5	1b ± 0.15
J-DEPI	0.7 ± 0.05c	ND	2.5 ± 0.19b	ND	3.2a ± 0.6	0.9b ± 0.4	4.4c ± 0.6	1.3a ± 0.4
J-QEPI	0.8 ± 0.06c	ND	2.6 ± 0.18a	ND	3.4a ± 0.6	0.9b ± 0.4	4.5b ± 0.6	1.1b ± 0.3

ND: not detected; TVC: total viable count; CBC: coliform bacterial counts. Mean ± SD. Means in the same column with different letters are significantly different (*p* ≤ 0.05).
